# Supplementary material for: Calculation of ATP production rates using the Seahorse XF Analyzer
Source: EMBO Rep. 2023 Aug 7;24(10):e56380. doi: 10.15252/embr.202256380 (PMC10561364; doi:10.15252/embr.202256380)
Supplement: Supplementary file 1 — Appendix [file EMBR-24-e56380-s006.docx]

**Table of Contents**

Appendix Table S1: Lactate:H^+^ values used for composite factor Page 2

Appendix Table S2: H^+^:O_2_ values used for composite factor Page 3

**Appendix Table S1: Lactate:H^+^ values used for composite factor**

| **Cell Type** | **Lactate:H+** |
| --- | --- |
| 3T3-L1 | 1.73 |
| A431 | 1.28 |
| A549 | 1.60 |
| BAEC | 1.58 |
| BMDM | 1.58 |
| BT474 | 1.51 |
| C2C12 | 1.46 |
| Cortical neuron | 1.45 |
| H460 | 1.58 |
| HCT116 | 1.43 |
| HepG2 | 1.36 |
| HUVEC | 1.55 |
| Jurkat | 1.54 |
| MCF10A | 1.64 |
| MDA-MB231 | 1.61 |
| PC12 | 1.74 |
| Raw 264.7 | 1.45 |
|  |  |
| **Average** | **1.53** |
| **St. Dev.** | **0.12** |

**Appendix Table S2: H^+^:O_2_ values used for composite factor**

| **Cell Type** | **H+/O2** |
| --- | --- |
| 3T3-L1 | 0.40 |
| A431 | 0.35 |
| A549 | 0.39 |
| BAEC | 0.34 |
| BMDM | 0.47 |
| C2C12 | 0.33 |
| Cortical astrocyte | 0.43 |
| Cortical neuron | 0.49 |
| H460 | 0.40 |
| HCT116 | 0.35 |
| HepG2 | 0.36 |
| HUVEC | 0.45 |
| INS-1 | 0.34 |
| Jurkat | 0.38 |
| MCF10a | 0.31 |
| MCF7 | 0.35 |
| MDA-MB231 | 0.38 |
| NRVM | 0.33 |
| PC12 | 0.41 |
| RAW 264.7 | 0.28 |
| Splenocytes | 0.35 |
|  |  |
| **AVERAGE** | **0.38** |
| **St. Dev.** | **0.05** |
