# Supplementary material for: Calculation of ATP production rates using the Seahorse XF Analyzer
Source: EMBO Rep. 2023 Aug 7;24(10):e56380. doi: 10.15252/embr.202256380 (PMC10561364; doi:10.15252/embr.202256380)
Supplement: Supplementary file 2 — Expanded View Figures PDF [file EMBR-24-e56380-s009.pdf]

Expanded View Figures

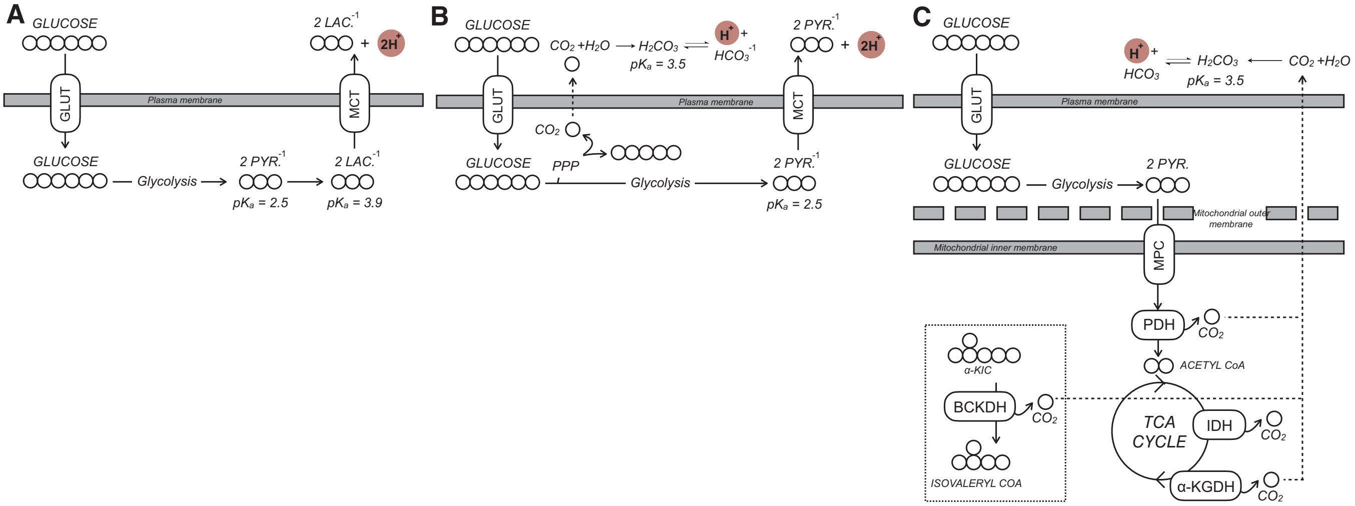

Figure EV1. Metabolic reactions that generate net pH changes.

A The main driver of extracellular acidification in most cell culture monolayer systems is lactate efflux. Uncharged glucose is taken up by the cell and converted to anionic pyruvate via glycolysis. Subsequent fermentation to lactate and efflux via monocarboxylate transporters causes a net pH change in the experimental medium that is detected by the XF Analyzer. GLUT, glucose transporter; MCT, monocarboxylate transporter; PYR., pyruvate; LAC., lactate.

B Other reactions can cause a net pH change in the experimental medium, such as the efflux of glucose-derived carbon as anionic pyruvate or CO_2 evolved from the oxidative pentose phosphate pathway (PPP) that ultimately forms bicarbonate and H^+.

C CO_2 evolution by mitochondrial dehydrogenases also generates bicarbonate that can be detected by ECAR. CO_2 is evolved from various mitochondrial enzymes including isocitrate dehydrogenase (IDH) and the three oxoacid dehydrogenases: pyruvate dehydrogenase (PDH), α-ketoglutarate dehydrogenase (α-KGDH), and the branched chain keto acid dehydrogenase (BCKDH). α-KIC, α-ketoisocaproic acid, the keto acid derivative of leucine.

 © 2023 The Authors

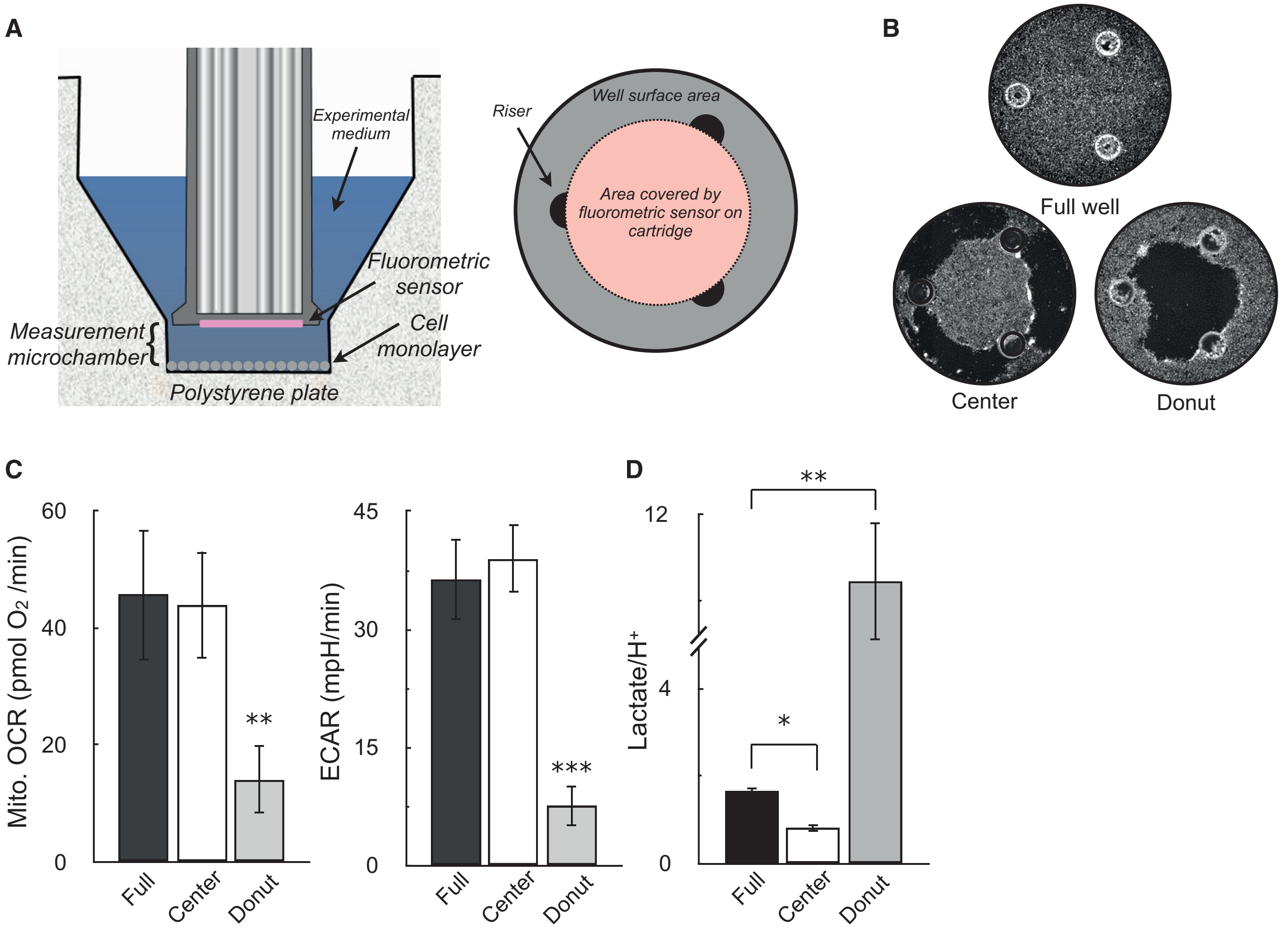

Figure EV2. Sensor coverage of the well plate can explain why lactate:$H^+ > 1$.

A (*Left*) Cutaway image of Seahorse XF microplate and measurement sensor depicting location of the measurement microchamber, cell monolayer, and fluorometric sensor attached to the measurement cartridge. (*Right*) Birds-eye view diagram of the XF microplate well, where the confluent monolayer in drawn in gray, the three plate risers maintaining the uniform volume of the measurement microchamber are drawn in black, and the area covered by the fluorometric sensor is given in pink.

B Representative images of wells assayed with an uninterrupted monolayer of cells ("Full well"), wells with cells scraped off the outer rim ("Center"), and wells with cells scraped off the inner portion of the well ("Donut").

C OCR and ECAR in A549 cells for the scraping conditions described in (B) ($n = 4$ biological replicates).

D The ratio of lactate (measured by enzymatic assay) to H^+ (measured by the XF Analyzer) for the conditions described in (A and B). Assay medium is as in Fig. 1C ($n = 4$ biological replicates).

All data are mean ± S.E.M. Statistical analysis was analyzed by a one-way, repeated measures ANOVA followed by Dunnett's *post hoc* multiple comparisons tests. *$P < 0.05$; **$P < 0.01$; ***$P < 0.001$.

© 2023 The Authors

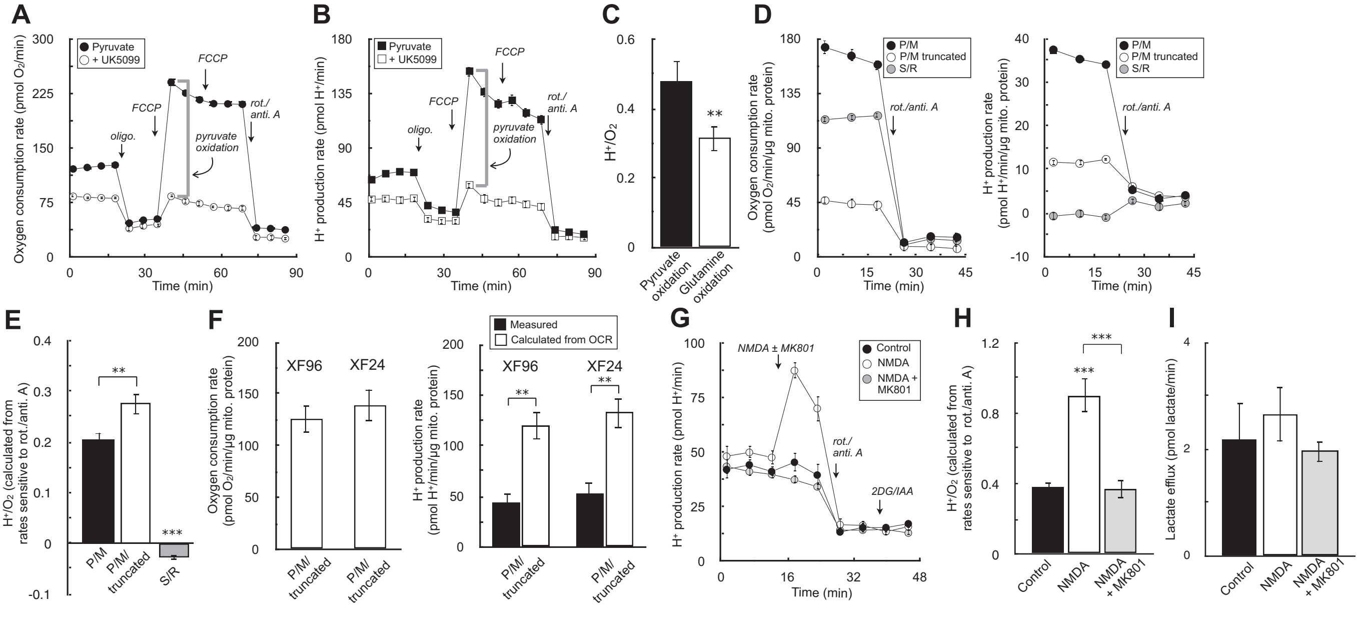

Figure EV3. Cellular substrate preference and non-glycolytic organic acid efflux can affect H^+:O_2 ratios.

A Sample oxygen consumption kinetic trace for HepG2 cells offered 5 mM pyruvate, 5 mM 2-deoxyglucose (2-DG), and 50 μM iodoacetate (IAA) in the experimental medium. Where indicated, cells were treated with 1 μM UK5099 for 20 min prior to measurements (n = 10 technical replicates).

B Sample H^+ production rates for the conditions as in (A) (n = 10 technical replicates).

C The H^+:O_2 ratio is given for pyruvate and glutamine oxidation. Rates of H^+ and O_2 production for pyruvate oxidation were calculated as the UK5099-sensitive, maximal rates of respiration & acidification as indicated by the gray brackets in (A and B). Rates for glutamine oxidation were calculated similarly except 5 mM glutamine was offered instead pyruvate in the experimental medium, and the glutaminase inhibitor CB-839 was used instead of UK5099 (n = 4 biological replicates).

D Sample kinetic traces of oxygen consumption (*left*) and H^+ production (*right*) for rat heart mitochondria offered 10 mM pyruvate with 1 mM malate and 2 mM dichloroacetate (P/M), a substrate and inhibitor mix to run a truncated TCA cycle consisting of P/M supplemented with 60 μM fluorocitrate (to block isocitrate dehydrogenase), 2 mM malonate (to block succinate dehydrogenase), and 1 mM aminooxyacetate (to block transaminase activity) (P/M truncated), or 10 mM succinate with 2 μM rotenone (S/R). All respiration measurements were made in the presence of 2 μM oligomycin and 4 μM FCCP, and background rates were calculated in response to 200 nM rotenone and 1 μM antimycin A (n = 20 technical replicates).

E The H^+:O_2 ratio is calculated for each condition as in (D) (n = 6 biological replicates).

F (*Left*) Oxygen consumption rates in rat heart mitochondria offered conditions as in (D) for P/M truncated assayed in both the XF24 and XF96 assay platforms (n = 5 biological replicates). (*Right*) Measured H^+ production rates for conditions as before (closed bars), compared to the theoretically predicted value assuming every mol of acid is stoichiometrically read by the instrument's H^+ sensor (open bars) (n = 5 biological replicates).

G Primary cortical neurons are offered 5 mM β-hydroxybutyrate in aCSF medium supplemented with 5 mM 2-DG, and 50 μM IAA. Where indicated, cells were acutely treated with 100 μM NMDA or NMDA plus the NMDA receptor inhibitor MK-801 (10 μM) (n = 10 technical replicates).

H The H^+:O_2 ratio is calculated for each condition as in (F) (n = 4 biological replicates).

I The lactate efflux rate as measured by enzymatic assay for neurons treated for 20 min with the conditions as in (F) (n = 3 biological replicates).

All data are mean ± S.E.M. Statistical analysis for EV3E, EV3H, and EV3I was analyzed by a one-way, repeated measures ANOVA followed by Dunnett's *post hoc* multiple comparisons tests. Individual pairwise comparisons for EV3C and EV3F were analyzed by a two-tailed Student's t-test. $^{**}P < 0.01$; $^{***}P < 0.001$.

 © 2023 The Authors

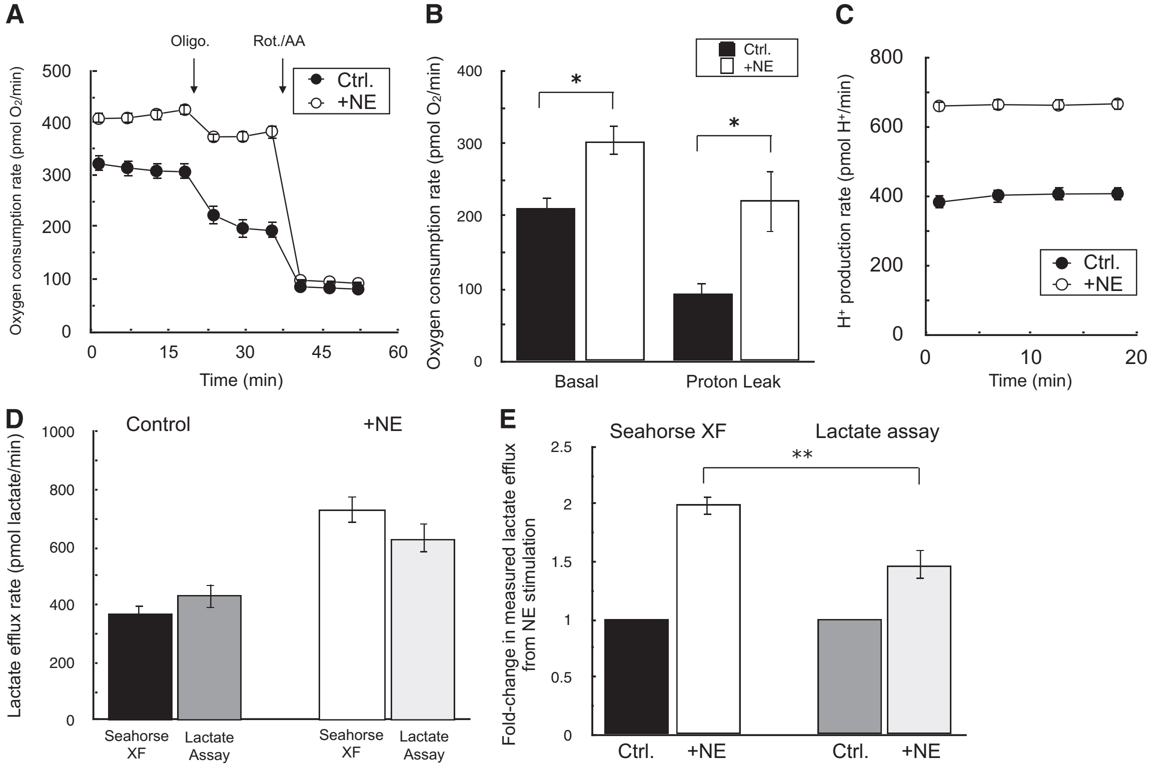

Figure EV4. Changes in proportion of organic acid efflux during cell activation.

All data are mean ± S.E.M. unless otherwise specified. *P < 0.05; **P < 0.01, ***P < 0.001.

- A Sample oxygen consumption kinetic trace for iBAT cells offered 10 mM glucose, 2 mM glutamine, and 2 mM pyruvate in the experimental medium. Where indicated, cells were treated with 2 μM oligomycin or 200 nM rotenone with 1 μM antimycin A. NE = 5 μM norepinephrine (*n* = 10 technical replicates).
- B Sample kinetic trace of the H^+ production rate as in (A) (*n* = 10 technical replicates).
- C Rates of basal mitochondrial respiration rates and respiration associated with proton leak for conditions as in (A) (*n* = 3 biological replicates).
- D Lactate efflux rates calculated by either the Seahorse XF Analyzer or an enzymatic lactate assay as described in the manuscript methods. NE, as before (*n* = 3 biological replicates).
- E Fold-change in lactate efflux in response to norepinephrine as measured by the Seahorse XF Analyzer or an enzymatic lactate assay. NE, as before (*n* = 3 biological replicates).

All data are mean ± S.E.M. Statistical analysis was analyzed by a two-tailed Student's *t*-test. *P < 0.05; **P < 0.01.

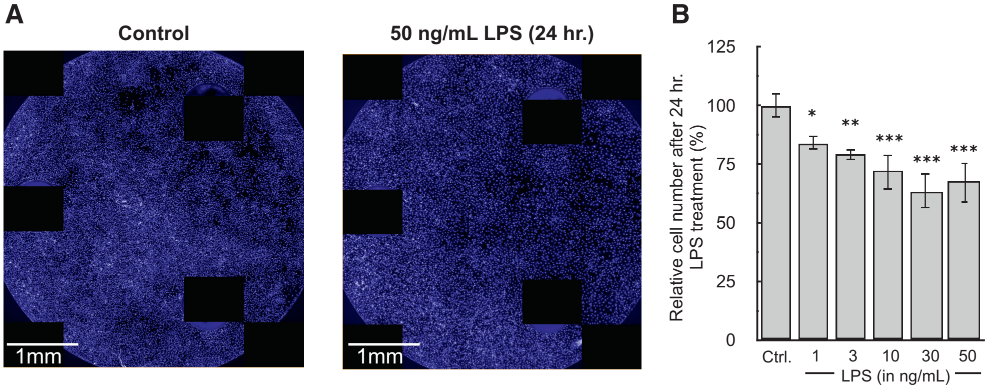

Figure EV5. Images of well plate after 24 h. LPS treatment.

A BMDMs were plated at 5 × 10^4 cells/well treated with 50 ng/ml LPS for 24 h. in Seahorse XF96 well plates. (A) After the assay, cells were fixed overnight with 2% (v/v) paraformaldehyde, stained with Hoescht 33342, and images were captured using the Perkin Elmer Operetta.

B Quantification of cell counts after 24 h. treatment with LPS (n = 4 biological replicates).

All data are mean ± S.E.M. Statistical analysis was analyzed by a one-way, repeated measures ANOVA followed by Dunnett's *post hoc* multiple comparisons tests. *$P < 0.05$; **$P < 0.01$; ***$P < 0.001$.

© 2023 The Authors
