# Supplementary material for: Calculation of ATP production rates using the Seahorse XF Analyzer
Source: EMBO Rep. 2023 Aug 7;24(10):e56380. doi: 10.15252/embr.202256380 (PMC10561364; doi:10.15252/embr.202256380)
Supplement: Supplementary file 14 [file EMBR-24-e56380-s013.pdf]

EMBO reports

Calculation of ATP production rates using the Seahorse XF Analyzer

Brandon Desousa, Kristen Kim, Anthony Jones, Andréa Ball, Wei Hsieh, Pamela Swain, Danielle Morrow, Alexandra Brownstein, David Ferrick, Orian Shirihai, Andrew Neilson, David Nathanson, George Rogers, Brian Dranka, Anne Murphy, Charles Affourtit, Steven J. Bensinger, Linsey Stiles, Natalia Romero, and Ajit Divakaruni
DOI: 10.15252/embr.202256380

Corresponding author(s): Ajit Divakaruni (ADivakaruni@mednet.ucla.edu)

Review Timeline:

Submission Date:	26th Oct 22
Editorial Decision:	9th Dec 22
Revision Received:	16th Mar 23
Editorial Decision:	26th Apr 23
Revision Received:	5th Jul 23
Accepted:	14th Jul 23

Editor: Ioannis Papaioannou

Transaction Report:

(Note: With the exception of the correction of typographical or spelling errors that could be a source of ambiguity, letters and reports are not edited. Depending on transfer agreements, referee reports obtained elsewhere may or may not be included in this compilation. Referee reports are anonymous unless the Referee chooses to sign their reports.)

Dear Ajit,

Thank you for submitting your manuscript for consideration by EMBO reports. It has been seen by three experts in the field, and I have already sent you a copy of their reports (appended again below).

I have asked you for feedback and we have discussed your proposed revision plan. Following our discussion, I would like to invite you to reframe your manuscript with the focus on the methodological aspects and to revise it along the lines you suggested (for further consideration as a "Methods & Resources" piece). Please note that the referee concerns (as detailed in their reports) must be fully addressed and their suggestions taken on board. Please address all referee concerns in a complete point-by-point response. Acceptance of the manuscript will depend on a positive outcome of a second round of review. It is EMBO reports policy to allow a single round of revision only and acceptance or rejection of the manuscript will therefore depend on the completeness of your responses included in the next, final version of the manuscript. If you have any questions or comments, we can also discuss the revisions in a video chat, if you like.

We realize that it is difficult to revise to a specific deadline. In the interest of protecting the conceptual advance provided by the work, we usually recommend a revision within 3 months (March 8th). Please discuss with me the revision progress ahead of this time if you require more time to complete the revisions.

*****
IMPORTANT NOTE:
We perform an initial quality control of all revised manuscripts before re-review. Your manuscript will FAIL this control and the handling will be DELAYED if the following APPLIES:

1) A data availability section providing access to data deposited in public databases is missing.

2) Your manuscript contains statistics and error bars based on n=2. Please use scatter plots in these cases. No statistics should be calculated if n=2.

When submitting your revised manuscript, please carefully review the instructions that follow below. Failure to include requested items will delay the evaluation of your revision.
*****

When submitting your revised manuscript, we will require:

1) a .docx formatted version of the manuscript text (including legends for main figures, EV figures and tables). Please make sure that the changes are highlighted to be clearly visible.

2) individual production quality figure files as .eps, .tif, .jpg (one file per figure).
Please download our Figure Preparation Guidelines (figure preparation pdf) from our Author Guidelines pages https://www.embopress.org/page/journal/14693178/authorguide for more info on how to prepare your figures.

3) a .docx formatted letter INCLUDING the reviewers' reports and your detailed point-by-point responses to their comments. As part of the EMBO Press transparent editorial process, the point-by-point response is part of the Review Process File (RPF), which will be published alongside your paper unless you opt out of this (please see below for further information).

4) a complete author checklist, which you can download from our author guidelines (<https://www.embopress.org/page/journal/14693178/authorguide>). Please insert information in the checklist that is also reflected in the manuscript. The completed author checklist will also be part of the RPF.

5) Please note that all corresponding authors are required to supply an ORCID ID for their name upon submission of a revised manuscript (<https://orcid.org/>). Please find instructions on how to link your ORCID ID to your account in our manuscript tracking system in our Author guidelines (<https://www.embopress.org/page/journal/14693178/authorguide#authorshipguidelines>)

6) We would kindly ask you to use "structured methods", our Materials and Methods format for "Methods & Resources" papers (see example: <http://msb.embopress.org/content/14/7/e8071>). The Materials and Methods section should include a Reagents and Tools Table (listing key reagents, experimental models, software and relevant equipment and including their sources and relevant identifiers) followed by a Methods and Protocols section in which methods can be described using a step-by-step protocol format with bullet points. More information is available at < https://www.embopress.org/page/journal/17444292/authorguide#methodsguide , paragraph "Structured Methods">.

7) We replaced Supplementary Information with Expanded View (EV) Figures and Tables that are collapsible/expandable online. A maximum of 5 EV Figures can be typeset. EV Figures should be cited as 'Figure EV1, Figure EV2" etc... in the text and their respective legends should be included in the main text after the legends of regular figures.

- For the figures that you do NOT wish to display as Expanded View figures, they should be bundled together with their legends in a single PDF file called *Appendix*, which should start with a short Table of Content. Appendix figures should be referred to in the main text as: "Appendix Figure S1, Appendix Figure S2" etc. See detailed instructions regarding expanded view here: <https://www.embopress.org/page/journal/14693178/authorguide#expandedview>

- Additional Tables/Datasets should be labeled and referred to as Table EV1, Dataset EV1, etc. Legends have to be provided in a separate tab in case of .xls files. Alternatively, the legend can be supplied as a separate text file (README) and zipped together with the Table/Dataset file.

8) Before submitting your revision, primary datasets (and computer code, where appropriate) produced in this study need to be deposited in an appropriate public database (see < https://www.embopress.org/page/journal/14693178/authorguide#dataavailability>).
Specifically, we would kindly ask you to provide public access to the following datasets/data:
- Mass spectrometry (GC/MS) data

Please remember to provide a reviewer password if the datasets are not yet public.

The accession numbers and database should be listed in a formal "Data Availability " section (placed after Materials and Methods) that follows the model below (see also < https://www.embopress.org/page/journal/14693178/authorguide#dataavailability>). Please note that the Data Availability Section is restricted to new primary data that are part of this study.

Data availability

The datasets (and computer code) produced in this study are available in the following databases:

- RNA-Seq data: Gene Expression Omnibus GSE46843 (https://www.ncbi.nlm.nih.gov/geo/query/acc.cgi?acc=GSE46843)
- [data type]: [name of the resource] [accession number/identifier/doi] ([URL or identifiers.org/DATABASE:ACCESSION])

*** Note - All links should resolve to a page where the data can be accessed. ***

9) We updated our journal's competing interests policy in January 2022 and request authors to consider both actual and perceived competing interests. Please review the new policy (<https://www.embopress.org/competing-interests>) and update your competing interests statement if necessary. Please name this section 'Disclosure and competing interests statement' and place it after the Acknowledgements section.

10) Figure legends and data quantification:
The following points must be specified in each figure legend:

- the name of the statistical test used to generate error bars and P values,
- the number (n) of independent experiments (please specify technical or biological replicates) underlying each data point,
- the nature of the bars and error bars (s.d., s.e.m.)
- If the data are obtained from n {less than or equal to} 2, use scatter plots showing the individual data points.

Discussion of statistical methodology can be reported in the materials and methods section, but figure legends should contain a basic description of n, P and the test applied.

See also the guidelines for figure legend preparation:
https://www.embopress.org/page/journal/14693178/authorguide#figureformat

11) We now request the publication of original source data with the aim of making primary data more accessible and transparent to the reader. Our source data coordinator will contact you to discuss which figure panels we would need source data for and will also provide you with helpful tips on how to upload and organize the files.

12) Our journal encourages inclusion of *data citations in the reference list* to directly cite datasets that were re-used and obtained from public databases. Data citations in the article text are distinct from normal bibliographical citations and should directly link to the database records from which the data can be accessed. In the main text, data citations are formatted as follows: "Data ref: Smith et al, 2001" or "Data ref: NCBI Sequence Read Archive PRJNA342805, 2017". In the Reference list, data citations must be labeled with "[DATASET]". A data reference must provide the database name, accession

number/identifiers and a resolvable link to the landing page from which the data can be accessed at the end of the reference. Further instructions are available at <https://www.embopress.org/page/journal/14693178/authorguide#referencesformat>.

13) Please also note our reference format:
<http://www.embopress.org/page/journal/14693178/authorguide#referencesformat>.

14) We now use CRediT to specify the contributions of each author in the journal submission system. CRediT replaces the author contribution section, which should be removed from the manuscript. Please use the free text box to provide more detailed descriptions. See also guide to authors:
<https://www.embopress.org/page/journal/14693178/authorguide#authorshipguidelines>.

15) As part of the EMBO publications' Transparent Editorial Process, EMBO reports publishes online a Review Process File to accompany accepted manuscripts. This File will be published in conjunction with your paper and will include the referee reports, your point-by-point response and all pertinent correspondence relating to the manuscript.

You can opt out of this by letting the editorial office know (emboreports@embo.org). If you do opt out, the Review Process File link will point to the following statement: "No Review Process File is available with this article, as the authors have chosen not to make the review process public in this case."

I look forward to seeing a revised version of your manuscript when it is ready. Please let me know if you have any questions or comments regarding the revision.

You can use this link to submit your revision: https://embor.msubmit.net/cgi-bin/main.plex

Best regards,

Ioannis

Ioannis Papaioannou, PhD
Editor
EMBO reports

Referee #1:

Metabolic switching between glycolysis and oxidative phosphorylation has emerged as a key step in a broad range of biological processes including fibrosis, inflammation and the immune response. This has occurred on the background of decades of metabolic research which is embedded in textbooks that compares glycolysis on mitochondrial oxphos solely on the basis of ATP production. While this mechanism does contribute to acute changes in energy demand the changes associated with cell differentiation are likely to be different. This excellent paper addresses this head-on and conclusively demonstrates that it is not just about the ATP. Minor comments below
1) I like the intro-it might be useful to comment that the absolute concentration of ATP in the cell is not a measure of energetic status. It is the turnover of ATP that is critical and if that flux is sufficient to maintain an appropriate ATP/ADP ratio then a cell is just as healthy as another irrespective of the amount of ATP.
2) In a similar vein it might be useful to mention Pi here. This is important since the delta G from ATP hydrolysis is dependent on ADP * Pi.

Referee #2:

This paper argues for the adequate quantification of ATP generation from oxidative phosphorylation versus glycolysis suing in vitro approaches. They specifically focus on macrophage activation-associated ATP demand. While i find the work of relevance and some novelty, the fact that the entire study relies on ex vivo analyses undermines the title of the paper and the potential translational value of the work. I suggest that the authors consider the study as a technical clarification/comment regarding the interpretation of data from in vitro analyses.

Other than those important issues, the paper has no physiological value for interpretation.

Referee #3:

In this paper, Desousa et al. describe how OCR and ECAR data from extracellular flux measurements can be used to quantify glycolytic and oxidative ATP production. The authors illustrate this data conversion process clearly and step-by-step, stating specific requirements in terms of experimental design that need to be met and pointing out potential pitfalls. Lastly, they showcase the virtues and importance of this method by monitoring dynamic and sometimes strikingly rapid changes in the source of ATP production and the overall ATP demand upon biological/pharmacological stimulation in several cell types, such as macrophages, neurons, and T cells.
Overall, this manuscript is well written and it provides an invaluable contribution to the field, because it explains how to fully capitalize on routine respirometry measurements, which are now conducted in almost every institute. However, as this publication may very likely serve as blueprint/guide for many future publications, a few things need to be addressed to make it even more comprehensible and usable for a broad audience.

Criticism:

#1 This manuscript pursues a balancing act between describing a novel methodology (calculating ATP production rates from Seahorse XF96 data) and answering a biological question (glycolytic vs. oxidative ATP production during pro-inflammatory macrophage activation). Starting at "line 434", it is not entirely clear why the authors haven chosen these examples or why they have sought to address exactly these biological questions. Was it because this particular area (immunometabolism) would benefit most from this publication, because previous publications relying on classical ATP endpoint determinations obviously came to wrong conclusions, or simply because immune cells show a very strong response to activation representing a convenient example to emphasize the usefulness of this method? If the focus of this manuscript is the methodological aspect, the authors may consider removing the last passage of the results part ("line 518 - line 550"), because the key message "oxidative phosphorylation and glycolysis are independently regulated during pro-inflammatory macrophage activation" is already substantiated in the preceding paragraph. However, if the shifts in immunometabolism upon stimulation were the key message, the manuscript would have to be re-evaluated.
Moreover, the title is quite vague, i.e. what topic does this publication cover, which cells were studied (mostly immune cells for the functional part), and what is activation?

#2 As already mentioned before, a broad readership may find this publication interesting, and thus not only experts in bioenergetics and mitochondrial biology but also scientists from other fields will most likely read this article. Consequently, not every reader will be familiar with basic knowledge about glycolysis, the TCA cycle, and the mitochondrial electron transport chain, which the authors take for granted. Adding a few explanatory sentences and elaborating slightly more on a few details, will make it easier for the reader to digest the wealth of information and to follow the authors' reasoning.
"Line 18" why does the method described here still detect an increased demand?
"Line 41" which factors can contaminate ECAR measurements and how?
"Line 304" how does lactate and respiratory CO2 contribute to acidification?
"Line 327" the inexperienced user may not know what a Seahorse cell culture microplate looks like. Therefore, adding a schematic representation of a fourth well (the bottom part of it), including the three spacers, the growth area covered by the sensor probe, and the growth area not covered by the sensor probe, could be very helpful to understand the discrepancy between calculated and enzymatically-determined lactate levels in the medium.
"Line 641" which factors are these and how would they contribute to underestimation of acidification?

The introductory sentence and the sentence summarizing the conclusion of each paragraph are sometimes too technical. The authors could try to simplify the objective and the outcome, which is obviously not an easy task without diluting the crux of the main message.

#3 The authors should provide a short list of the most important "dos and don'ts" when calculating ATP production rates from OCR and ECAR data. Moreover, the experimental requirements (treatment has to precede the addition of oligomycin, choice of injection chemicals and order of injections, cell types to avoid), which allow/preclude quantification of ATP production, should be explicitly stated.
In this context, would the authors agree that any given treatment that causes a release of metabolites, which may act as weak organic acids, into the assay medium would contaminate ECAR readings? Apart from the example included in the manuscript (NMDA-induced efflux of glutamate), adipocytes release large amounts of fatty acids into the medium during active lipolysis. Is this method still applicable to (stimulated) adipocytes?

#4 It should be emphasized that this method was developed with a Seahorse XF96/e/pro analyzer and that it cannot be applied to data obtained with Seahorse XF24 devices.

#5 Would the authors advise users to always validate calculated lactate efflux rates with an enzymatic assay first?

#6 The abstract or the introduction should include a short section explaining the advantages of this method, why it is superior to commonly applied methods, and why scientists should use it (in very simple language).

#7 "Line 72" there is no such thing as a C57BL/6 mouse (C57BL/6J vs. C57BL/6N).

We thank the reviewers for the constructive feedback and are heartened by the largely positive comments. Below we provide detailed responses to the suggestions, with the original reviewer comments in red, indented italics. The comments were invaluable in helping us refine and refocus our piece, and we did our best to constructively address each comment to make our piece more useful for a general audience.

Referee #1:

Metabolic switching between glycolysis and oxidative phosphorylation has emerged as a key step in a broad range of biological processes including fibrosis, inflammation and the immune response. This has occurred on the background of decades of metabolic research which is embedded in textbooks that compares glycolysis on mitochondrial oxphos solely on the basis of ATP production. While this mechanism does contribute to acute changes in energy demand the changes associated with cell differentiation are likely to be different. This excellent paper addresses this head-on and conclusively demonstrates that it is not just about the ATP. Minor comments below

We thank the reviewer for their positive comments, particularly calling the piece an 'excellent paper.' We are also grateful that our perspective that metabolism is not simply about ATP production – and that flux through specific metabolic pathways can adjust cell function and fate – was able to come through despite the manuscript being focused mostly on bioenergetic methods.

1) I like the intro-it might be useful to comment that the absolute concentration of ATP in the cell is not a measure of energetic status. It is the turnover of ATP that is critical and if that flux is sufficient to maintain an appropriate ATP/ADP ratio then a cell is just as healthy as another irrespective of the amount of ATP.
*2) In a similar vein it might be useful to mention Pi here. This is important since the delta G from ATP hydrolysis is dependent on ADP * Pi.*

Both of these are excellent points. We have incorporated the suggestions into the introduction in **Lines 46-49** of the revised manuscript.

Lines 46-49:

"During physiological cell activation, cells can readily increase the rate of ATP production to match the increased rate of consumption without appreciably changing steady-state ATP levels. A far more useful metric is the ATP:ADP ratio, reflecting the free energy change associated with ATP hydrolysis into ADP and inorganic phosphate"

Referee #2:

This paper argues for the adequate quantification of ATP generation from oxidative phosphorylation versus glycolysis suing in vitro approaches. They specifically focus on macrophage activation-associated ATP demand. While i find the work of relevance and some novelty, the fact that the entire study relies on ex vivo analyses undermines the title of the paper and the potential translational value of the work. I suggest that the authors consider the study as a technical clarification/comment regarding the interpretation of data from in vitro analyses.

Other than those important issues, the paper has no physiological value for interpretation.

We are most thankful for the reviewer's suggestion to reframe the piece as a methods-focused resource rather than a traditional research paper. Candidly, we struggled with whether to frame the work as a methods piece with the various experiments as proofs-of-concept, or a research manuscript enabled

by the outlined methods. In retrospect, we entirely agree with the reviewer to focus on the technical advance and reframe the experimental conclusions.

Referee #3:

In this paper, Desousa et al. describe how OCR and ECAR data from extracellular flux measurements can be used to quantify glycolytic and oxidative ATP production. The authors illustrate this data conversion process clearly and step-by-step, stating specific requirements in terms of experimental design that need to be met and pointing out potential pitfalls. Lastly, they showcase the virtues and importance of this method by monitoring dynamic and sometimes strikingly rapid changes in the source of ATP production and the overall ATP demand upon biological/pharmacological stimulation in several cell types, such as macrophages, neurons, and T cells.
Overall, this manuscript is well written and it provides an invaluable contribution to the field, because it explains how to fully capitalize on routine respirometry measurements, which are now conducted in almost every institute. However, as this publication may very likely serve as blueprint/guide for many future publications, a few things need to be addressed to make it even more comprehensible and usable for a broad audience.

We are beyond grateful to have had a such a knowledgeable reviewer read the paper with this level of depth and seriousness, and with the objective of making the paper more useful to a broad readership. As we hope was clear, our goal is indeed to improve the accuracy of the glycolytic measurements, and as such expand the utility of the Seahorse XF Analyzer. The perspective of the reviewer was therefore extraordinarily helpful in helping us reframe the manuscript, pulling it out of the proverbial 'weeds' to help make it more accessible and friendly to its intended audience.

We are pleased that a scientist of this caliber found the piece 'well written' and an 'invaluable contribution to the field,' and have done our best to faithfully incorporate every suggestion as detailed below to make the manuscript better.

Criticism:

#1 This manuscript pursues a balancing act between describing a novel methodology (calculating ATP production rates from Seahorse XF96 data) and answering a biological question (glycolytic vs. oxidative ATP production during pro-inflammatory macrophage activation). Starting at "line 434", it is not entirely clear why the authors haven chosen these examples or why they have sought to address exactly these biological questions. Was it because this particular area (immunometabolism) would benefit most from this publication, because previous publications relying on classical ATP endpoint determinations obviously came to wrong conclusions, or simply because immune cells show a very strong response to activation representing a convenient example to emphasize the usefulness of this method? If the focus of this manuscript is the methodological aspect, the authors may consider removing the last passage of the results part ("line 518 - line 550"), because the key message "oxidative phosphorylation and glycolysis are independently regulated during pro-inflammatory macrophage activation" is already substantiated in the preceding paragraph. However, if the shifts in immunometabolism upon stimulation were the key message, the manuscript would have to be re-evaluated.

Moreover, the title is quite vague, i.e. what topic does this publication cover, which cells were studied (mostly immune cells for the functional part), and what is activation?

It is heartening to learn the reviewer understood our difficulty in the 'balancing act' between framing the manuscript as either a methods piece with the various experiments as proofs-of-concept, or a research manuscript enabled by the outlined methods. As discussed in the response to Reviewer #2, we agree we missed the mark with our earlier submission, and have entirely reframed the manuscript as a 'Methods and Resources' piece.

We now focus the manuscript on clearly outlining the method and its utility to the non-specialist, and explicitly frame the experiments as application of the method using various models of cell activation. To that point, we have removed what was previously Figure #7 (MyD88-associated TLR activation enhancing glycolysis independently of respiration). As a methods-focused resource, immunometabolic shifts themselves are no longer a focus, and LPS-induced changes in macrophage metabolism (now Fig. 5) are simply presented alongside the glioblastoma (Fig. 4) and neuronal and T cell activation assays (now Fig. 6) as proofs-of-concept of metabolic shifts upon cell activation or adaptation.

#2 As already mentioned before, a broad readership may find this publication interesting, and thus not only experts in bioenergetics and mitochondrial biology but also scientists from other fields will most likely read this article. Consequently, not every reader will be familiar with basic knowledge about glycolysis, the TCA cycle, and the mitochondrial electron transport chain, which the authors take for granted. Adding a few explanatory sentences and elaborating slightly more on a few details, will make it easier for the reader to digest the wealth of information and to follow the authors' reasoning.

We thank the reviewer for this perspective to – on balance – write the piece geared toward the broad group of non-experts that could benefit from this manuscript rather than the experts that can understand every nuance and detail. As a result, the entire manuscript is restructured, as detailed in the subsequent comments. Some major changes include:

- The introduction has been almost entirely rewritten to be more geared towards the general audience (**Lines 17-90**) with an introductory paragraph explaining the basics of energy metabolism (**Lines 18-29**).

- Figure EV1 now pictorially represents how lactate as well as additional sources of acidification contribute to the ECAR readings.

Expanded View Figure 1

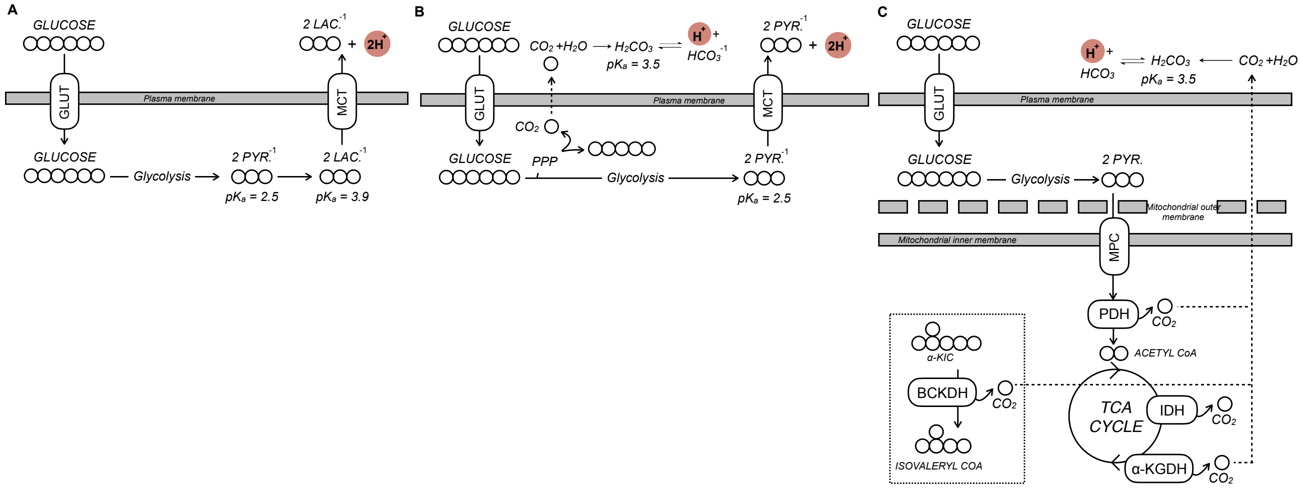

- The detailed experiments described in Figures 1-3 (**Lines 295-512** of the revised manuscript) are now written in a more structured & outlined format to help users follow exactly what hypothesis is being tested and why.

"Line 18" why does the method described here still detect an increased demand?

Although this line has now been removed from the manuscript due to restructuring the introduction, we describe this principle differently in **Lines 46-48** of the revised manuscript.

Lines 46-48:

"During physiological cell activation, cells can readily increase the rate of ATP production to match the increased rate of consumption without appreciably changing steady-state ATP levels."

"Line 41" which factors can contaminate ECAR measurements and how?

In addition to Fig. EV1 shown earlier, we now address this in **Lines 69-79** of the Introduction.

Lines 69-79:

"Additionally, ECAR measurements can be influenced by cellular processes other than lactate efflux that also result in a net acidification of the extracellular medium. Indeed, the main component of ECAR in two-dimensional cell culture systems is often glycolysis and fermentation, reflecting the uptake of uncharged glucose and the release of anionic lactate. However, other acidifying reactions can contribute to the measured ECAR. For example, CO_2 evolution during oxidative metabolism (e.g. dehydrogenases of the TCA cycle or the pentose phosphate pathway) acidifies the medium after hydration and generation of bicarbonate ($CO_2 + H_2O \rightarrow H_2CO_3 \rightarrow H^+ + HCO^{3-}$). Additionally, specific cell types can release appreciable amounts of organic acids such as pyruvate, glutamate, and short-chain fatty acids that may be reflected in ECAR measurements. As such, the extracellular acidification rate cannot itself quantify lactate efflux."

"Line 304" how does lactate and respiratory CO2 contribute to acidification?

This has now been addressed in the Introduction and Figure EV1.

"Line 327" the inexperienced user may not know what a Seahorse cell culture microplate looks like. Therefore, adding a schematic representation of a fourth well (the bottom part of it), including the three spacers, the growth area covered by the sensor probe, and the growth area not covered by the sensor probe, could be very helpful to understand the discrepancy between calculated and enzymatically-determined lactate levels in the medium.

This is an excellent suggestion, and we once again are grateful for this perspective of keeping the novice user in mind. We have included these schemes in Fig. EV2A, which is copied below:

A

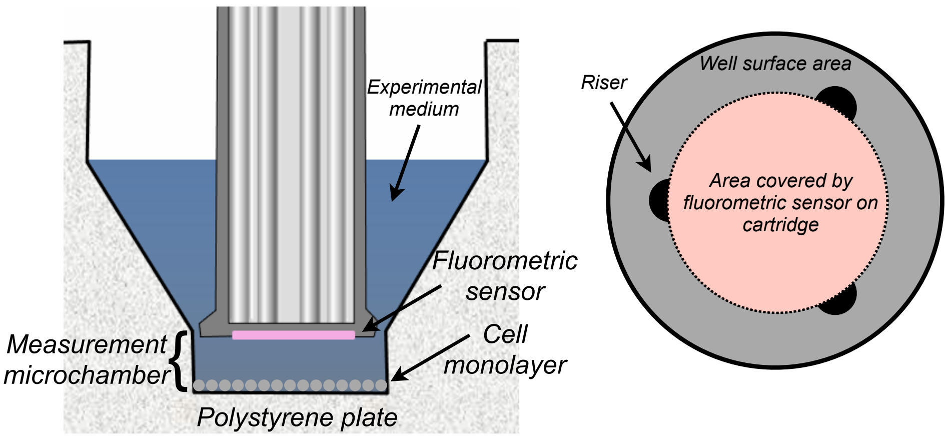

"Line 641" which factors are these and how would they contribute to underestimation of acidification?

This was a good suggestion to explicitly call out that we were referring to how material unseen by the measurement sensor causes the underestimation of the rate. This is now corrected in **Lines 689-690** of the revised manuscript.

The introductory sentence and the sentence summarizing the conclusion of each paragraph are sometimes too technical. The authors could try to simplify the objective and the outcome, which is obviously not an easy task without diluting the crux of the main message.

We thank the reviewer for this suggestion. We now try to begin every introductory paragraph and results/discussion section with a sentence or two explaining the overall idea or the goal of a given experiment. This way, the reader can logically follow the story even if the contents of the paragraph or section is necessarily in-depth given the rigor required to validate this method. Writing in this style was very helpful in gearing the manuscript to a broad audience, and we will adopt this style going forward for other manuscripts even beyond this technical piece.

#3 The authors should provide a short list of the most important "dos and don'ts" when calculating ATP production rates from OCR and ECAR data. Moreover, the experimental requirements (treatment has to precede the addition of oligomycin, choice of injection chemicals and order of injections, cell types to avoid), which allow/preclude quantification of ATP production, should be explicitly stated.

This a fantastic recommendation, and one that we should have thought to include in our original submission. The manuscript discussion now ends with a “Best practices” section in **Lines 721-751** of our revised manuscript. The text is too long to reproduce here, but we thank the reviewer for this suggestion that has improved our manuscript.

In this context, would the authors agree that any given treatment that causes a release of metabolites, which may act as weak organic acids, into the assay medium would contaminate ECAR readings? Apart from the example included in the manuscript (NMDA-induced efflux of glutamate), adipocytes release large amounts of fatty acids into the medium during active lipolysis. Is this method still applicable to (stimulated) adipocytes?

We thank the reviewer for suggesting that we address this important question regarding what happens when activation itself changes the composition of organic acid efflux. We experimentally tested this in immortalized human brown adipocytes activated with noradrenaline in Figure EV4 **Lines 499-512**. of the revised manuscript describe the Figure, and the overall message in **Lines 506-512** are reproduced here along with Figure E4V itself. Additionally, the implications of this result are addressed in the “Best Practices” section of the manuscript Discussion as detailed in Comment #5.

Lines 506-512:

“As before, comparing lactate efflux calculated from the XF Analyzer against that measured from an enzymatic assay was within experimental error of multiple biological replicates (Fig. EV4D). However, it was clear that non-glycolytic acid production was increased upon norepinephrine stimulation: the XF Analyzer showed almost a two-fold increase in lactate production in response to adrenergic activation, while the enzymatic assay measured only a 1.5-fold increase (Fig. EV4E). This suggests that H^+ measurements were somewhat confounded by efflux of organic acids distinct from lactate.”

Expanded View Figure 4

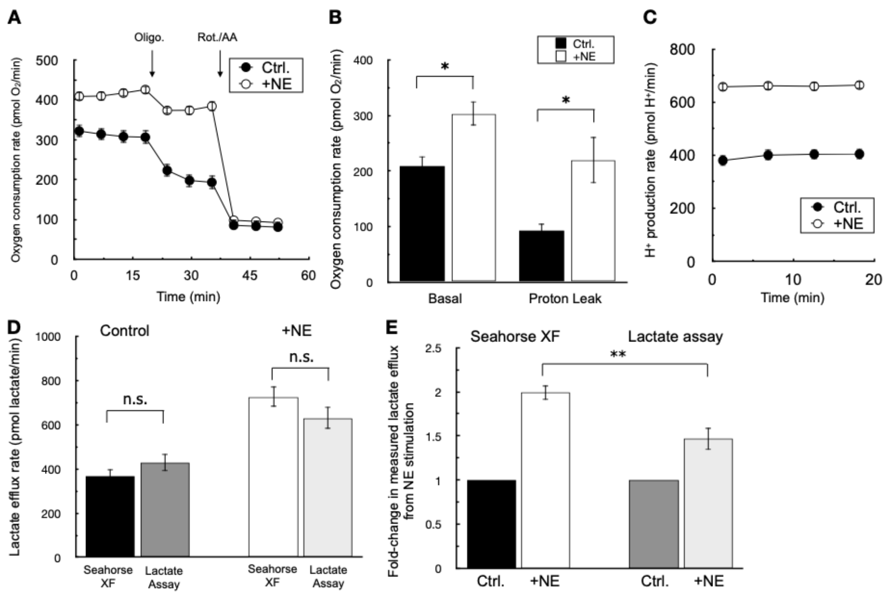

#4 It should be emphasized that this method was developed with a Seahorse XF96/e/pro analyzer and that it cannot be applied to data obtained with Seahorse XF24 devices.

This an excellent suggestion, and we now explicitly state this in **Lines 314-317** of the revised manuscript.

Lines 314-317:

"The work presented uses a 96-well Seahorse XF Analyzer platform. Although some values cannot be directly translated to 24-well instruments due to differences in volume of the measurement microchamber, the experimental strategy to calculate these values is universal across all Seahorse XF platforms.

However, we do write that while some values themselves cannot be directly applied, the same basic principles for calculating lactate:H^+ and H^+:O_2 are universal across instrument platforms. In fact, we now include Figure EV3F, showing that the XF96 and XF24 instruments indentically handle respiratory acidification from isolated mitochondria. The data is described in **Lines 420-424** of the revised manuscript.

#5 Would the authors advise users to always validate calculated lactate efflux rates with an enzymatic assay first?

This is an important question, and we apologize for not explicitly addressing this in the earlier version of the manuscript. The extensive validation data presented in Fig. 1, Fig. 3A, and Fig. 4A all run lactate assays alongside XF data to provide reassurance that enzymatic assays need not be run under most conditions. **Lines 456-458** of the revised manuscript now read:

Lines 456-458 (after describing Fig. 3A):

"Taken together with Figure 1, the results suggest the consensus values and approximations used here should be applicable across a broad range of cell types under normal assay conditions.

However, as discussed in Comment #3, it is clearly the case that the approach is less accurate under conditions where the rate of lactate efflux is matched by efflux of other organic acids. This is therefore addressed in the "Best Practices" section of the Discussion in **Lines 735-751** of the revised manuscript.

Lines 735-751:

"Additionally, it may be that the approximations used for this method are substantively inaccurate in specific cases depending on the experimental hypothesis and quantitative rigor required. For example, rates of lactate efflux may be particularly low when using acutely isolated primary cells, and therefore the non-zero background rate of H^+ efflux (Figs. 1E and 2C) may represent a substantial component of the signal and skew results. Furthermore, conditions where the rate of lactate efflux is matched by organic acid efflux of the same magnitude may also be less amenable to using consensus values of lactate:H^+ and H^+:O_2. Indeed, this is apparent in noradrenaline-stimulated adipocytes known to release fatty acids: our XF Analyzer calculations based on H^+ release estimated an almost two-fold increase in lactate efflux, whereas the enzymatic assay showed a 1.5-fold increase (Fig. EV4). Additionally, under extreme, non-physiological conditions such as neurons depolarized in the absence of glucose, release of glutamate and other neurotransmitters resulted in a profound increase in acidification entirely independent of lactate efflux."

"As such, any system in which the investigator believes that non-lactate acid efflux could prohibitively alter the conclusions should consider independently calculating lactate efflux with other methods such as enzymology or mass spectrometry. However, the empirical approach presented here provides the framework for researchers to calculate lactate:H^+ and H^+:O_2 values tailored to any (monolayer) model system or experimental conditions."

#6 The abstract or the introduction should include a short section explaining the advantages of this method, why it is superior to commonly applied methods, and why scientists should use it (in very simple language).

This an excellent suggestion, and we now reinforce the advantages of the method in both the abstract and the introduction.

Lines 12-15:

"This method generates a single readout that allows the direct comparison of ATP produced from oxidative phosphorylation and glycolysis in live cells. Additionally, the manuscript provides a framework for tailoring the calculations to specific cell systems or experimental conditions."

Lines 80-86:

"We therefore developed a method to transform OCR and ECAR into rates of ATP production from oxidative phosphorylation and glycolysis. Although measurements of OCR and ECAR have become a central cell biology technique, the qualitative nature of the analysis makes it difficult to discriminate between healthy, physiological shifts in bioenergetic pathways or compensatory responses due to mitochondrial dysfunction. This method detailed here provides a solution to this challenge in a single, live cell readout estimating the total rate of cellular ATP production as well as its distribution between oxidative phosphorylation and glycolysis."

#7 "Line 72" there is no such thing as a C57BL/6 mouse (C57BL/6J vs. C57BL/6N).

We thank the reviewer for pointing this out. Of course, this was a lack of attention to detail on our part, and all mice used were C57BL/6J from Jackson Laboratories (000664). This is now corrected in **Line 100** of the revised manuscript.

1st Revision - Editorial Decision 26th Apr 2023

Dear Ajit,

Thank you for the submission of your revised manuscript to EMBO reports. We have now received the full set of reports from the three referees that were asked to re-evaluate your study, and I have already sent you a copy of their comments (included again below).

The referees find the revised version significantly improved and they now all recommend publication. There is only one minor clarification request from referee #2, which should be addressed in the next version of the manuscript (please use change tracking in Word).

From the editorial side, there are also a few things that we need from you:

- Your manuscript will be published under the Methods and Resources track. We would therefore kindly ask you to use structured methods, our new "Materials and Methods" format, which is mandatory for Methods papers (see example: <http://msb.embopress.org/content/14/7/e8071>). The "Materials and Methods" section should include a "Reagents and Tools table" (listing key reagents, experimental models, software and relevant equipment, and including their sources and relevant identifiers) followed by a "Methods and Protocols" section in which methods can be described using a step-by-step protocol format with bullet points. More information is available at <https://www.embopress.org/page/journal/17444292/authorguide#methodsguide>.

- The abstract should be written in present tense, and it should not exceed 175 words.

- Please provide up to 5 keywords in your revised manuscript.

- Please note that a data availability statement is mandatory. If your study does not include any datasets requiring deposition in a public database, please add the statement: "This study includes no data deposited in external repositories." under the heading "Data availability" at the end of Materials and Methods.

- Please update your competing interests statement: the heading should be "Disclosure and competing interests statement".

- We noticed a discrepancy in the name of a co-author: "Brandon R. Desousa" in the manuscript vs. "Brandon Desosua" in the online submission system. Please correct it.

- The author contributions statement should be removed from the manuscript file. Instead, we now use CRediT to specify the contributions of each author in the journal submission system. Please use the free text box to provide more detailed descriptions. See also our guide to authors: <https://www.embopress.org/page/journal/14693178/authorguide#authorshipguidelines>.

- You are kindly requested to note our reference format and update your list accordingly (<http://www.embopress.org/page/journal/14693178/authorguide#referencesformat>). References need to be alphabetical (e.g. Van den Bossche comes under B and not V in the current version of the manuscript) and "et al." is needed after 10 names.

- According to our journal's policy, "data not shown" (stated on page 9 of your manuscript) is not permitted. All data referred to in the paper should be displayed in the main or Expanded View figures, or in the Appendix. Please add these data or change the text accordingly if these data are not central to the study and its conclusions.

- Please update your author checklist: for each relevant entry, the last column ("In which section is the information available?" should be completed.

- Please provide all relevant funding information both in the Acknowledgements section of the revised manuscript and in the online submission system (grant number 995337 is missing in the manuscript; Eugene V. Cota Robles Scholarship is missing in our online system).

- The Appendix Table callouts need correcting to "Appendix Table S#".

- Please upload your "Appendix Worksheet" tabs as separate Expanded View Datasets, using the nomenclature "Dataset EV#", in individual ZIP files, and update the callouts in the manuscript accordingly. Each ZIP file should contain the data (Excel) file AND a separate plain text README file with the item title and description. Please submit these using the file type Expanded View File in our manuscript submission system.

- Please include a Table of Contents (with page numbers) on the first page of your Appendix.

- Please note that EMBO press papers are accompanied online by:
A) a short (1-2 sentences) summary of the findings and their significance,
B) 2-4 short bullet points highlighting the key results, and
C) a synopsis image that is exactly 550 pixels wide and 300-600 pixels high (the height is variable). You can either show a model or key data in the synopsis image. Please note that the text needs to be readable at the final size.
Please send us this information along with your revised manuscript (the text for A and B should be provided in a separate Word file).

- Our source data coordinator has already contacted you with instructions on the source data that need to be uploaded. Please include them in the submission of your revised manuscript.

- The Expanded View Figure legends need to be in the manuscript Word file (at the end of the manuscript).

- The manuscript sections are in the wrong order. Please follow the order of this example: (<http://msb.embopress.org/content/14/7/e8071>).

- Your Figure legends have been inspected by our data editors for completeness and accuracy. Please see the required changes in the attached Word file and address all comments in your revised manuscript (with tracked changes).

Please also note that as part of the EMBO publications' Transparent Editorial Process, EMBO reports publishes online a Review Process File to accompany accepted manuscripts. This File will be published in conjunction with your paper and will include the referee reports, your point-by-point response and all pertinent correspondence relating to the manuscript.

You can opt out of this by letting the editorial office know (emboreports@embo.org). If you do opt out, the Review Process File link will point to the following statement: "No Review Process File is available with this article, as the authors have chosen not to make the review process public in this case."

We would also welcome the submission of cover suggestions or motifs to be used by our Graphics Illustrator in designing a cover.

We look forward to seeing a final version of your manuscript as soon as possible. Please use this link to submit your revision: https://embor.msubmit.net/cgi-bin/main.plex

Best regards,

Ioannis

Ioannis Papaioannou, PhD
Editor
EMBO reports

Referee #1:

Very responsive to the points raised. An excellent contribution which will be very valuable to the community

----
Referee #2:

In this interesting work, Dr. Brandon R. Desousa and his colleagues demonstrated that is possible to use oxygen consumption and extracellular acidification rates in a Seahorse experiment to calculate the ATP production rate and the contribution of glycolysis and OXPHOS in the ATP production. The group was capable to demonstrate step-by-step how to perform the calculation and rise all the pitfalls and limitations of this new approach to calculating ATP production.
Although it is clear to the authors which seahorse assay was performed, Mitostress or Glycostress, to collect the oxygen consumption rate (OCR) and extracellular acidification rate (ECAR), to apply in the equation. It is not clear in the current form of the manuscript which assay must be used to collect those variables, and which specific time to collect.

POINT-BY-POINT REVIEWER RESPONSE

Please find below a point-by-point response to both the reviewer comments as well as the editorial suggestions/changes for manuscript EMBOR-2022-56380V2 (Desousa BR et al. "Calculation of ATP production rates using the Seahorse XF Analyzer.")

Reviewer comments:

> *Reviewer 2: "…Although it is clear to the authors which seahorse assay was performed, Mitostress or Glycostress, to collect the oxygen consumption rate (OCR) and extracellular acidification rate (ECAR), to apply in the equation. It is not clear in the current form of the manuscript which assay must be used to collect those variables, and which specific time to collect."*

We now explicitly address this in the preamble to the step-by-step protocol. We are hesitant to call out the terms "Mitostress" or "Glycostress" as they refer to pre-packaged, commercial kits which are not at all necessary to conduct the measurements and can confuse the novice user. However, in addition to the existing figures, text, and Expanded View Datasets that detail the calculations and conditions in painstaking detail, we have added the following text (**Lines 767-771**):

> "These calculations are amenable to standard protocols for respirometry profiling (i.e. measurements of OCR and ECAR in response to sequential additions of oligomycin, FCCP, and rotenone with antimycin A.). The measurement times at which data points are collected follow standard best practices (Divakaruni et al., 2014; Divakaruni and Jastroch, 2022) and examples are provided in the Expanded View Datasets EV1-EV4."

Editorial comments:

> *(1) Your manuscript will be published under the Methods and Resources track. We would therefore kindly ask you to use structured methods, our new "Materials and Methods" format, which is mandatory for Methods papers*

We sincerely apologize, as we had done this for the initial revision V1 but erroneously put this information into a separate file uploaded as "Protocol and Resource Table." We have used the manuscript provided (Trepte et al.; DOI:10.15252/msb.20178071) as suggested to guide our reformatting. In the revised V2 manuscript, the Resources and Tools Table is now included in the Materials and Methods in the main text (beginning **Line 558)**, and a step-by-step protocol is given (**Lines 763-774**). As Seahorse XF Analysis is ubiquitous in metabolic research, and ample protocols for standardized assays are available, we have focused our Resource Table and step-by-step protocol specifically on the novel aspects advanced by this manuscript, namely the calculation of buffer powering power of the medium, correction of ECAR to reflect lactate efflux, and calculation of ATP production rates from the assay.

As we note in the preamble to the protocol (**Lines 764-769**): "The protocol provided here is written alongside the Expanded View Datasets EV1-EV4 for the calculation of ATP production rates. Established, time-tested protocols are available for conducting standardized respirometry assays with the Seahorse XF Analyzer (Pelletier et al., 2014) as well as guidelines for best practices (Divakaruni et al., 2014; Divakaruni and Jastroch, 2022). As such, this protocol focuses specifically on the calculation of the buffering power of the experimental medium and subsequent ATP production rates from a Seahorse XF assay."

(2) The abstract should be written in present tense, and it should not exceed 175 words.

We now describe the data in the abstract in the present tense, as indicated in the highlighted words in **Lines 7, 9, and 10**. The word count for the manuscript is 169 words.

(3) Please provide up to 5 keywords in your revised manuscript.

The following keywords have been added: oxidative phosphorylation, glycolysis, ATP, Seahorse XF Analyzer, ECAR. These are found on the highlighted on the abstract page (**Page 2; Lines 19-20**).

(4) Data availability statement

As requested, a statement at the end of the Materials and Methods section (**Lines 847-848**) now reads:

"Data availability: This study includes no data deposited in external repositories."

(5) Update competing interests statement

As requested, the heading for this section now reads "Disclosure and competing interests statement" (**Line 858**)

(6) Discrepancy in Brandon Desousa's name

Thank you for bringing this inconsistency to our attention – this has been corrected in the online submission system to include his middle initial R. as the preferred name is "Brandon R. Desousa"

(7) Author contribution statement

The author contributions statement has now been removed and the CRediT system in the online submission portal now represents the author contributions.

(8) Reference formatting

Thank you for bringing this to our attention. We previously used the Mendeley Reference Manager with the "EMBO Reports" style, but it appears the style was not updated after the shift to the Harvard style. We have shifted to Harvard style references and manually curated the references to match recent EMBO Reports manuscripts (unfortunately we did not use EndNote and could not use the provided plugin). As requested, we note that "Van den Bossche et al." is now properly alphabetized and references with more than 10 authors are noted with *et al* as requested.

(9) Use of "data not shown"

We apologize for the use of the phrase "data not shown" and have removed this entirely. The statement was in reference to an unnecessary, ancillary point and is of no relevance to the conclusions of the manuscript.

(10) Updated author checklist

The author checklist has been updated to include information in the last column regarding where the information is located. We apologize for neglecting this in the earlier submission.

(11) Funding acknowledgements

Thank you for the note to update funding acknowledgements in accordance with the online submission system. The grant # for the W.M. Keck Foundation grant has been added to the manuscript text. Additionally, the support from the Cota Robles Scholarship has been removed, as after consultation with colleagues and my administration, I learned this funding stream is generally not acknowledged in manuscripts and there is no need to do so.

(12) Appendix Table Callouts

The Appendix Tables are now called out as “Appendix Table S1” and “Appendix Table S2” as suggested.

(13) Reformat Appendix Worksheet Tabs to separate Expanded View datasets

This has now been done as outlined in the directions to the best of our understanding. The individual Excel tabs from the previous “Appendix Worksheet” are now referred to as Datasets EV1-EV4. Zipped files containing a README.txt file describing the data along with each Expanded View Dataset have been uploaded as per instructions.

(14) Appendix Table of Contents

A Table of Contents has been added to the Appendix Tables

(15) Summary of findings, bullet points, and synopsis image

These files have been provided with the revised ‘V2’ iteration of the manuscript.

(16) Source data

The source data have been provided as per instructions (to the best of our ability & understanding) in a Zipped folder along with README.txt files describing each figure panel. We have also completed the Source Data checklist accordingly.

(17) Expanded View legends in manuscript body

Thank you kindly for pointing this out and for the editorial staff helping us with this, having cut-and-pasted this information already. We have approved/accepted the sections in the revised text.

(18) Manuscript sections are out of order

We have followed the instructions accordingly and now have the Materials and Methods section after the Discussion.

(19) Provide additional information in figure legends

This has now been done according to the comments left by the editorial staff and all questions answered.

(20) Transparent editorial process

We would be happy to make public the editorial process for the manuscript, as it has been a thoroughly enjoyable experience.

2nd Revision - Editorial Decision 14th Jul 2023

Prof. Ajit Divakaruni
University of California, Los Angeles
Department of Molecular and Medical Pharmacology;
650 Charles E Young Dr. S
Los Angeles, CA 90095
United States

Dear Ajit,

I am very pleased to accept your manuscript for publication in the next available issue of EMBO reports. Thank you for your contribution to our journal.

At the end of this email I include important information about how to proceed. Please ensure that you take the time to read the information and complete and return the necessary forms to allow us to publish your manuscript as quickly as possible.

As part of the EMBO publication's Transparent Editorial Process, EMBO reports publishes online a Review Process File to accompany accepted manuscripts. As you are aware, this File will be published in conjunction with your paper and will include the referee reports, your point-by-point response and all pertinent correspondence relating to the manuscript.

If you do NOT want this File to be published, please inform the editorial office within 2 days, if you have not done so already, otherwise the File will be published by default [contact: emboreports@embo.org]. If you do opt out, the Review Process File link will point to the following statement: "No Review Process File is available with this article, as the authors have chosen not to make the review process public in this case."

Thank you again for your contribution to EMBO reports and congratulations on a successful publication. Please consider us again in the future for your most exciting work.

Best regards,

Ioannis

Ioannis Papaioannou, PhD
Editor
EMBO reports

****

THINGS TO DO NOW:

Please note that you will be contacted by Wiley Author Services to complete licensing and payment information. The required 'Page Charges Authorization Form' is available here: https://www.embopress.org/pb-assets/embo-site/er_apc.pdf - please download and complete the form and return to embopressproduction@wiley.com

EMBO Press participates in many Publish and Read agreements that allow authors to publish Open Access with reduced/no publication charges. Check your eligibility: https://authorservices.wiley.com/author-resources/Journal-Authors/open-access/affiliation-policies-payments/index.html

You will receive proofs by e-mail approximately 2-3 weeks after all relevant files have been sent to our Production Office; you should return your corrections within 2 days of receiving the proofs.

Please inform us if there is likely to be any difficulty in reaching you at the above address at that time. Failure to meet our deadlines may result in a delay of publication, or publication without your corrections.

All further communications concerning your paper should quote reference number EMBOR-2022-56380V3 and be addressed to emboreports@wiley.com.

Should you be planning a Press Release on your article, please get in contact with emboreports@wiley.com as early as possible, in order to coordinate publication and release dates.

EMBO Press Author Checklist

Corresponding Author Name: Ajit Divakaruni
Journal Submitted to: EMBO Reports
Manuscript Number: EMBOR-2022-56380V1

USEFUL LINKS FOR COMPLETING THIS FORM
The EMBO Journal - Author Guidelines
EMBO Reports - Author Guidelines
Molecular Systems Biology - Author Guidelines
EMBO Molecular Medicine - Author Guidelines

Reporting Checklist for Life Science Articles (updated January 2022)
This checklist is adapted from Materials Design Analysis Reporting (MDAR) Checklist for Authors. MDAR establishes a minimum set of requirements in transparent reporting in the life sciences (see Statement of Task: 10.31222/osf.io/9sm4x). Please follow the journal's guidelines in preparing your manuscript.
Please note that a copy of this checklist will be published alongside your article.

Abridged guidelines for figures
1. Data
The data shown in figures should satisfy the following conditions:
- the data were obtained and processed according to the field's best practice and are presented to reflect the results of the experiments in an accurate and unbiased manner.
- ideally, figure panels should include only measurements that are directly comparable to each other and obtained with the same assay.
- plots include clearly labeled error bars for independent experiments and sample sizes. Unless justified, error bars should not be shown for technical replicates.
- if n<5, the individual data points from each experiment should be plotted. Any statistical test employed should be justified.
- Source Data should be included to report the data underlying figures according to the guidelines set out in the authorship guidelines on Data Presentation.

2. Captions
Each figure caption should contain the following information, for each panel where they are relevant:
- a specification of the experimental system investigated (eg cell line, species name).
- the assay(s) and method(s) used to carry out the reported observations and measurements.
- an explicit mention of the biological and chemical entity(ies) that are being measured.
- an explicit mention of the biological and chemical entity(ies) that are altered/varied/perturbed in a controlled manner.
- the exact sample size (n) for each experimental group/condition, given as a number, not a range;
- a description of the sample collection allowing the reader to understand whether the samples represent technical or biological replicates (including how many animals, litters, cultures, etc.).
- a statement of how many times the experiment shown was independently replicated in the laboratory.
- definitions of statistical methods and measures:
  - common tests, such as t-test (please specify whether paired vs. unpaired), simple χ2 tests, Wilcoxon and Mann-Whitney tests, can be unambiguously identified by name only, but more complex techniques should be described in the methods section;
  - are tests one-sided or two-sided?
  - are there adjustments for multiple comparisons?
  - exact statistical test results, e.g., P values = x but not P values < x;
  - definition of 'center values' as median or average;
  - definition of error bars as s.d. or s.e.m.

Please complete ALL of the questions below.
Select "Not Applicable" only when the requested information is not relevant for your study.

Materials

Newly Created Materials	Information included in the manuscript?	In which section is the information available? (Reagents and Tools Table, Materials and Methods, Figures, Data Availability Section)
New materials and reagents need to be available; do any restrictions apply?	Not Applicable	

Antibodies	Information included in the manuscript?	In which section is the information available? (Reagents and Tools Table, Materials and Methods, Figures, Data Availability Section)
For **antibodies** provide the following information:<br>- Commercial antibodies: RRID (if possible) or supplier name, catalogue number and or/clone number<br>- Non-commercial: RRID or citation	Not Applicable	

DNA and RNA sequences	Information included in the manuscript?	In which section is the information available? (Reagents and Tools Table, Materials and Methods, Figures, Data Availability Section)
**Short novel DNA or RNA including primers, probes**: provide the sequences.	Not Applicable	

Cell materials	Information included in the manuscript?	In which section is the information available? (Reagents and Tools Table, Materials and Methods, Figures, Data Availability Section)
**Cell lines:** Provide species information, strain. Provide accession number in repository **OR** supplier name, catalog number, clone number, and/**OR** RRID.	Yes	Materials and Methods
**Primary cultures:** Provide species, strain, sex of origin, genetic modification status.	Yes	Materials and Methods
Report if the cell lines were recently **authenticated** (e.g., by STR profiling) and tested for mycoplasma contamination.	Not Applicable	

Experimental animals	Information included in the manuscript?	In which section is the information available? (Reagents and Tools Table, Materials and Methods, Figures, Data Availability Section)
**Laboratory animals or Model organisms:** Provide species, strain, sex, age, genetic modification status. Provide accession number in repository **OR** supplier name, catalog number, clone number, **OR** RRID.	Yes	Materials and Methods
**Animal observed in or captured from the field:** Provide species, sex, and age where possible.	Not Applicable	
Please detail **housing and husbandry conditions**.	Not Applicable	

Plants and microbes	Information included in the manuscript?	In which section is the information available? (Reagents and Tools Table, Materials and Methods, Figures, Data Availability Section)
**Plants:** provide species and strain, ecotype and cultivar where relevant, unique accession number if available, and source (including location for collected wild specimens).	Not Applicable	
**Microbes:** provide species and strain, unique accession number if available, and source.	Not Applicable	

Human research participants	Information included in the manuscript?	In which section is the information available? (Reagents and Tools Table, Materials and Methods, Figures, Data Availability Section)
If collected and within the bounds of privacy constraints report on age, sex and gender or ethnicity for all study participants.	Not Applicable	

Core facilities	Information included in the manuscript?	In which section is the information available? (Reagents and Tools Table, Materials and Methods, Figures, Data Availability Section)
If your work benefited from core facilities, was their service mentioned in the acknowledgments section?	Not Applicable	

Design

Study protocol	Information included in the manuscript?	In which section is the information available? (Reagents and Tools Table, Materials and Methods, Figures, Data Availability Section)
If study protocol has been **pre-registered, provide DOI in the manuscript**. For clinical trials, provide the trial registration number **OR** cite DOI.	Not Applicable	
Report the **clinical trial registration number** (at ClinicalTrials.gov or equivalent), where applicable.	Not Applicable	

Laboratory protocol	Information included in the manuscript?	In which section is the information available? (Reagents and Tools Table, Materials and Methods, Figures, Data Availability Section)
Provide DOI OR other citation details if **external detailed step-by-step protocols** are available.	Not Applicable	

Experimental study design and statistics	Information included in the manuscript?	In which section is the information available? (Reagents and Tools Table, Materials and Methods, Figures, Data Availability Section)
Include a statement about **sample size** estimate even if no statistical methods were used.	Not Applicable	
Were any steps taken to minimize the effects of subjective bias when allocating animals/samples to treatment (e.g. **randomization procedure**)? If yes, have they been described?	Not Applicable	
Include a statement about **blinding** even if no blinding was done.	Not Applicable	
Describe **inclusion/exclusion criteria** if samples or animals were excluded from the analysis. Were the criteria pre-established?<br><br>If sample or data points were omitted from analysis, report if this was due to attrition or intentional exclusion and provide justification.	Not Applicable	
For every figure, are **statistical tests** justified as appropriate? Do the data meet the assumptions of the tests (e.g., normal distribution)? Describe any methods used to assess it. Is there an estimate of variation within each group of data? Is the variance similar between the groups that are being statistically compared?	Yes	Materials and Methods as well as Figure Legends

Sample definition and in-laboratory replication	Information included in the manuscript?	In which section is the information available? (Reagents and Tools Table, Materials and Methods, Figures, Data Availability Section)
In the figure legends: state number of times the experiment was **replicated** in laboratory.	Yes	Figure Legends
In the figure legends: define whether data describe **technical or biological replicates**.	Yes	Figure Legends

Ethics

Ethics	Information included in the manuscript?	In which section is the information available? (Reagents and Tools Table, Materials and Methods, Figures, Data Availability Section)
Studies involving **human participants**: State details of **authority granting ethics approval** (IRB or equivalent committee(s), provide reference number for approval.	Not Applicable	
Studies involving **human participants**: Include a statement confirming that **informed consent** was obtained from all subjects and that the experiments conformed to the principles set out in the WMA Declaration of Helsinki and the Department of Health and Human Services Belmont Report.	Not Applicable	
Studies involving **human participants:** For publication of **patient photos**, include a statement confirming that consent to publish was obtained.	Not Applicable	
Studies involving experimental **animals**: State details of **authority granting ethics approval** (IRB or equivalent committee(s), provide reference number for approval. Include a statement of compliance with ethical regulations.	Yes	Materials and Methods
Studies involving **specimen and field samples:** State if relevant **permits** obtained, provide details of authority approving study; if none were required, explain why.	Not Applicable	

Dual Use Research of Concern (DURC)	Information included in the manuscript?	In which section is the information available? (Reagents and Tools Table, Materials and Methods, Figures, Data Availability Section)
Could your study fall under dual use research restrictions? Please check biosecurity documents and list of **select agents and toxins** (CDC): https://www.selectagents.gov/sat/list.htm	Not Applicable	
If you used a select agent, is the security level of the lab appropriate and reported in the manuscript?	Not Applicable	
If a study is subject to dual use research of concern regulations, is the name of the **authority granting approval and reference number** for the regulatory approval provided in the manuscript?	Not Applicable	

Reporting

The MDAR framework recommends adoption of discipline-specific guidelines, established and endorsed through community initiatives. Journals have their own policy about requiring specific guidelines and recommendations to complement MDAR.

Adherence to community standards	Information included in the manuscript?	In which section is the information available? (Reagents and Tools Table, Materials and Methods, Figures, Data Availability Section)
State if relevant guidelines or checklists (e.g., **ICMJE, MIBBI, ARRIVE, PRISMA**) have been followed or provided.	Not Applicable	
For **tumor marker prognostic studies,** we recommend that you follow the **REMARK** reporting guidelines (see link list at top right). See author guidelines, under 'Reporting Guidelines'. Please confirm you have followed these guidelines.	Not Applicable	
For **phase II and III randomized controlled trials**, please refer to the **CONSORT** flow diagram (see link list at top right) and submit the CONSORT checklist (see link list at top right) with your submission. See author guidelines, under 'Reporting Guidelines'. Please confirm you have submitted this list.	Not Applicable	

Data Availability

Data availability	Information included in the manuscript?	In which section is the information available? (Reagents and Tools Table, Materials and Methods, Figures, Data Availability Section)
Have **primary datasets** been deposited according to the journal's guidelines (see 'Data Deposition' section) and the respective accession numbers provided in the Data Availability Section?	Not Applicable	
Were **human clinical and genomic datasets** deposited in a public access-controlled repository in accordance to ethical obligations to the patients and to the applicable consent agreement?	Not Applicable	
Are **computational models** that are central and integral to a study available without restrictions in a machine-readable form? Were the relevant accession numbers or links provided?	Not Applicable	
If publicly available data were reused, provide the respective **data citations in the reference list**.	Not Applicable	
